# Anti-Tuberculosis Drug Resistance among New and Previously Treated Sputum Smear-Positive Tuberculosis Patients in Uganda: Results of the First National Survey

**DOI:** 10.1371/journal.pone.0070763

**Published:** 2013-08-01

**Authors:** Deus Lukoye, Francis Adatu, Kenneth Musisi, George William Kasule, Willy Were, Rosemary Odeke, Julius Namonyo Kalamya, Ann Awor, Anand Date, Moses L. Joloba

**Affiliations:** 1 National Tuberculosis/Leprosy Program Ministry of Health, Kampala, Uganda; 2 Makerere University College of Health Sciences, Kampala, Uganda; 3 National Tuberculosis Reference Laboratory, Kampala, Uganda; 4 US Centers for Disease Control and Prevention, Division of Global HIV/AIDS, Atlanta, Georgia, United States of America; University of Witwatersrand, South Africa

## Abstract

**Background:**

Multidrug resistant and extensively drug resistant tuberculosis (TB) have become major threats to control of tuberculosis globally. The rates of anti-TB drug resistance in Uganda are not known. We conducted a national drug resistance survey to investigate the levels and patterns of resistance to first and second line anti-TB drugs among new and previously treated sputum smear-positive TB cases.

**Methods:**

Sputum samples were collected from a nationally representative sample of new and previously treated sputum smear-positive TB patients registered at TB diagnostic centers during December 2009 to February 2011 using a weighted cluster sampling method. Culture and drug susceptibility testing was performed at the national TB reference laboratory.

**Results:**

A total of 1537 patients (1397 new and 140 previously treated) were enrolled in the survey from 44 health facilities. HIV test result and complete drug susceptibility testing (DST) results were available for 1524 (96.8%) and 1325 (85.9%) patients, respectively. Of the 1209 isolates from new cases, resistance to any anti-TB drug was 10.3%, 5% were resistant to isoniazid, 1.9% to rifampicin, and 1.4% were multi drug resistant. Among the 116 isolates from previously treated cases, the prevalence of resistance was 25.9%, 23.3%, 12.1% and 12.1% respectively. Of the 1524 patients who had HIV testing 469 (30.7%) tested positive. There was no association between anti-TB drug resistance (including MDR) and HIV infection.

**Conclusion:**

The prevalence of anti-TB drug resistance among new patients in Uganda is low relative to WHO estimates. The higher levels of MDR-TB (12.1%) and resistance to any drug (25.3%) among previously treated patients raises concerns about the quality of directly observed therapy (DOT) and adherence to treatment. This calls for strengthening existing TB control measures, especially DOT, routine DST among the previously treated TB patients or periodic drug resistance surveys, to prevent and monitor development and transmission of drug resistant TB.

## Introduction

Tuberculosis (TB) remains one of the world’s leading causes of adult morbidity and mortality resulting in an estimated 8.8 million incident cases and 1.4 million deaths in 2010. Ninety-two percent of the cases occur in low and middle-income countries. Sub-Saharan Africa (SSA), a region with highest incidence of TB in the world hosts nine of the highest TB incidence countries globally [1]. The STOP TB strategy developed by the World Health Organization (WHO) aims to dramatically reduce the global burden of tuberculosis by 2015 by ensuring that all TB patients benefit from universal access to high-quality diagnosis and patient-centered treatment [2]. The HIV epidemic and the emergence of drug- resistant TB pose a serious challenge to achieving these ambitious goals. Treatment of multidrug resistant TB (MDR-TB) which is TB occurring in patients with strains of *Mycobacterium tuberculosis* resistant to at least rifampicin and isoniazid, was estimated to cost almost 30–40 times more than treatment of drug-sensitive disease in a recent study done in South Africa. In addition MDR-TB requires longer treatment with more toxic drugs, poorer treatment success rates, prolonged periods of morbidity and higher mortality as compared to drug sensitive TB [3], [4].

MDR-TB is gaining global importance with an estimated 440,000 cases occurring annually, representing about 3.6% of all TB cases across the world [5], [6]. Inappropriate drug regimens, non-adherence to treatment, transmission in congregate settings, substandard drug quality, and erratic drug supply are the major risk factors for development of drug resistant TB [7]. Mortality rates among MDR-TB patients have been reported to be as high as 37% and 89% among HIV-negative and HIV-positive patients respectively [8], [9]. Anti-TB drug resistance surveillance using routine drug susceptibility testing (DST) for all TB patients prior to starting their TB treatment would be ideal for monitoring the performance of TB control programs. However, due to the lack of routine DST services in most high TB prevalent countries, periodic surveys of representative samples of TB patients in the country are the only available source of information on the prevalence of anti-TB drug resistance. Despite the importance of these periodic surveys, the most recent WHO reports show that only 22 of the 46 countries in the African region have conducted these anti-TB drug resistance surveys [10]. Some studies have shown an association between HIV infection with rifampicin monoresistance [11]and MDR-TB outbreaks have been associated with HIV, although evidence showing HIV as an established independent risk factor for MDR is not yet documented [12]. The emergence of extensively drug resistant (XDR) TB, that is MDR-TB strains resistant to any fluoroquinolone and at least one of three injectable second-line drugs (i.e, amikacin, kanamycin, or capreomycin) and its association with high mortality among people living with HIV has raised a new challenge for TB control [13].

Uganda with an estimated population of 33 million ranks 19^th^ among the 22 high-TB burden countries in the world with an estimated incidence of 209/100,000 for all forms of TB [1]. About 8% of all the notified cases have had previous exposure to anti-TB drugs (relapses, defaulters or treatment failures). According to the WHO global report 2011, the cure rate was 31%, treatment completion 48%, death 8%, treatment failure 1% and treatment default 12%, among previously treated sputum smear-positive patients started on treatment. While among new patients, 28% were cured, 42% completed treatment, 5% died, 1% failed, 11% defaulted and approximately 14% were not evaluated.

Since 1997 Uganda has been using an eight-month regimen with two months of isoniazid, rifampicin pyrazinamide and ethambutol, followed by six months of isoniazid and ethambutol. For previously treated sputum smear-positive TB patients, the treatment regimen is two months of streptomycin, rifampicin, ethambutol, isoniazid and pyrazinamide, one month of rifampicin, ethambutol, isoniazid and pyrazinamide and 5 months of rifampicin, isoniazid and ethambutol. The mainstay of TB treatment in Uganda is community-based directly observed treatment (DOT). The National TB Leprosy Program initiated routine anti-TB drug resistance surveillance among re-treatment cases in 2008 although this has not been adequately implemented and improvement is still needed.

Limited anti-TB drug resistance surveys have been conducted so far, one in 1996–97 as part of global drug resistance surveillance that covered 3 zones. Two of the studies included new TB patients where the prevalence of MDR-TB was found to be 0.5% and 1.1% respectively [14]–[16]. Data on national anti-TB drug resistance rates and patterns in Uganda do not exist. The present study is the first national anti-tuberculosis drug resistance survey in Uganda conducted in accordance with the WHO-recommended methodology [17]. The objectives of this survey were to establish the prevalence of anti-TB drug resistance among new and previously treated smear positive TB patients and to assess the risk for anti-TB drug resistance among HIV-infected TB patients in the country.

## Methods

### Study Design

We obtained ethical approval from the ethical board at the Makerere University College of Health Sciences, the Uganda National Council of Science and technology, and Associate Director for Science at the United States, Centers for Disease Control & Prevention. All adult patients gave written informed consent before enrollment. Patients below 18 years assented and their consent was provided by guardians/parents.

### Sampling

A cluster sampling method was used in which 30 clusters (primary sampling units) were selected randomly with probability proportional to the number of smear-positive TB patients registered in 2005. Within each cluster a fixed number of consecutively diagnosed smear-positive patients were enrolled so that all included patients had identical sampling probabilities (“self-weighted sampling design”) [18]. Four hundred and ninety eight public health facilities in Uganda had TB diagnostic and treatment centers in 2007. We used data reported in these facilities to determine the average number of TB patients diagnosed per facility. The sample size was based on the number of new sputum-smear positive TB cases notified through the National TB and Leprosy Control Program (NTLP) in 2007 (n = 20,559) and designed to detect an assumed rifampicin prevalence of 1.4% [15] with 1% absolute precision for a 95% confidence interval (CI). Assuming a design effect of 2, estimated losses due to contamination and negative cultures of 15%, the final sample size was 1500 new sputum smear-positive patients with each cluster required to enroll 50 patients within a year.

**Figure 1:**
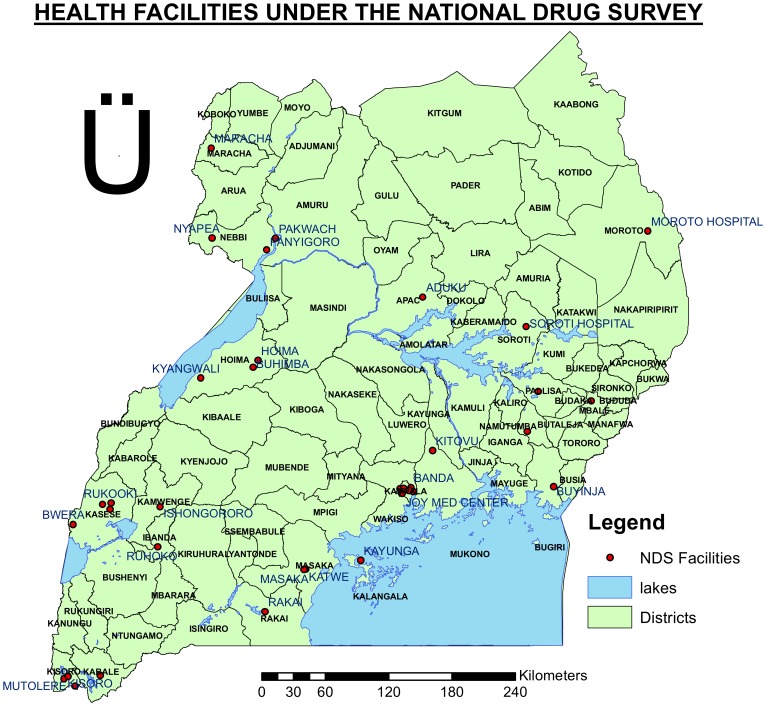
Map showing Health care facilties which participated in the National anti-TB drug resistance survey December 2009–February 2011.

A cluster was defined as a health care facility that was able to meet the requirement of 50 new smear-positive TB cases in a year (according to the 2007 enrollment). Where a facility was noted to have achieved enrollment of less than 50 new cases, it was merged with others depending on proximity to each other to have a group that was able to enroll the required minimum number of 50 cases. Such a group was called a pseudo-cluster. Clusters and pseudo-clusters were then listed. Based on the cumulative total enrollment 30 clusters/pseudo-clusters were selected randomly with probability proportional to the number of smear positive TB patients in accordance with the WHO guidelines [18]. Participants were enrolled from 44 diagnostic facilities [fig. 1]. Of these 21 were clusters involving 18 facilities (one had four clusters) and 9 were pseudo clusters involving 26 facilities. TB patients who were already on anti-TB treatment at the beginning of the study were excluded and enrollment of eligible patients into the study was done alongside provision of other services involved in treatment initiation including registration of patients in the unit TB registers for care. Consecutive eligible and consenting patients were enrolled in the survey until the sample size for each cluster was met. Alongside enrollment of new cases, all sputum- smear positive previously treated TB cases identified at the selected health facilities during this period were also included in the survey. Health care workers used a detailed questionnaire to collect demographic and clinical information to accurately classify patients as new or previously treated. Prior to the start of the survey, staff from all the selected health facilities were trained on the survey procedures and data instruments and participated in the piloting of instruments. A national coordination team was established to oversee and implement the survey.

### Data Collection

A standard clinical form was used to obtain data on patients’ demographic characteristics, HIV status prior to enrollment and previous history of TB treatment through a structured interview. In addition data about risk factors for exposure to resistant strains including imprisonment and those related to the patients’ social environment were collected. All TB patients at the sites including those eligible for enrollment were counseled and tested for HIV under routine conditions as required by the Uganda NTLP guidelines [19] and their results were included in the information sent on the case report form.

The national coordination team independently carried out re-interviews on patients randomly selected from the enrolled patients within 2 months of the original interview to validate their treatment history in order to allocate each patient to the correct category based on previous treatment.

### Laboratory Methods

#### Sputum collection &transportation

Each eligible patient who consented provided two sputum samples, an early morning and spot sample, independent of the routine samples used for diagnostic purposes to minimize chances of contamination of samples collected for the survey. No decontaminants were added. Samples were refrigerated at 4°C and then transported to the National TB Reference Laboratory (NTRL) for processing via a local courier system. Sputum samples were accompanied by a sputum shipment form that contained information about the date of sputum collection, participant number, and laboratory serial number and quantified results of sputum smear examination from the local laboratory.

#### Sputum culture and drug Susceptibility Testing (DST)

At the NTRL, samples were decontaminated using 1.5% NaOH NALC method. One of the samples, preferably an early morning sample was processed while the other was kept as a backup. The backup sample was analyzed if the first sample turned out as either negative or contaminated. The other sample was inoculated on 2 slopes of egg based Lowenstein-Jensen (L-J) medium, incubated at a temperature of 37°C and monitored weekly for growth up to 8 weeks. A culture was only reported negative if no growth was shown after 8 weeks. For the positive cultures identification of *M. tuberculosis* was done based on presumptive phenotypic appearance of colonies on the medium, and confirmed using insertion sequence 6110-based PCR method as previously described [20].

Isolates were tested for resistance to rifampicin, isoniazid, ethambutol and streptomycin using the L-J proportional method, in concentrations of 40 µg/ml for rifampicin, 0.2 µg/ml for isoniazid, 2.0 µg/ml for ethambutol and 4.0 µg/ml for streptomycin and all identified MDR-TB isolates were tested for resistance to kanamycin and ofloxacin using the same method in concentrations of 30 µg/ml & 2.0 µg/ml respectively.

We sent all rifampicin resistant isolates, a random sample of isolates from retreatment patients that were susceptible to isoniazid and rifampicin (n = 20) and a random sample of isoniazid resistant isolates sensitive to rifampicin (n = 20) to the supra-national reference laboratory (Borstel -Germany) for blinded external quality assurance.

### Definitions

A smear positive case in the study was defined as an individual in which at least one sputum sample was positive, for acid fast bacilli by direct Ziehl Neelsen staining. We defined a new patient as one who had not received first line anti-TB drugs for more than one month and previously treated if the patient had received first line anti-TB treatment for more than one month.

An MDR-TB patient was defined as one whose sputum isolate showed resistance to at least isoniazid and rifampicin while XDR-TB was defined as an MDR-TB patient whose isolate demonstrated resistance to kanamycin (as an injectable second line anti-TB drug) and ofloxacin (as a fluoroquinolone).

### Data Management

Data were double entered in epi-info V6, and discrepancies were corrected using the raw data. Analysis was done in Stata v.10 (Stata/Corp. College Station TX USA/.) For comparison of categorical variables we used the Chi-square test or the 2-sided Fishers’ exact test where appropriate. Multivariate analysis was done using logistic regression. We did all significance testing at 5% confidence level.

The outcome was the proportion of patients with drug resistance stratified by history of previous treatment calculated as a proportion across all clusters after weighing for the exact sampling probabilities for each new individual patient for whom DST results were available. These sampling weights were calculated as (number of patients in the cluster with DST results/50).In all these calculations confidence intervals and p-values were adjusted for cluster design by first-order Taylor linearization and by second-order correlation of Rao and Scott of the Pearson X^2^ respectively, as implemented by Stata svy commands. [21].

## Results

**Figure 2:**
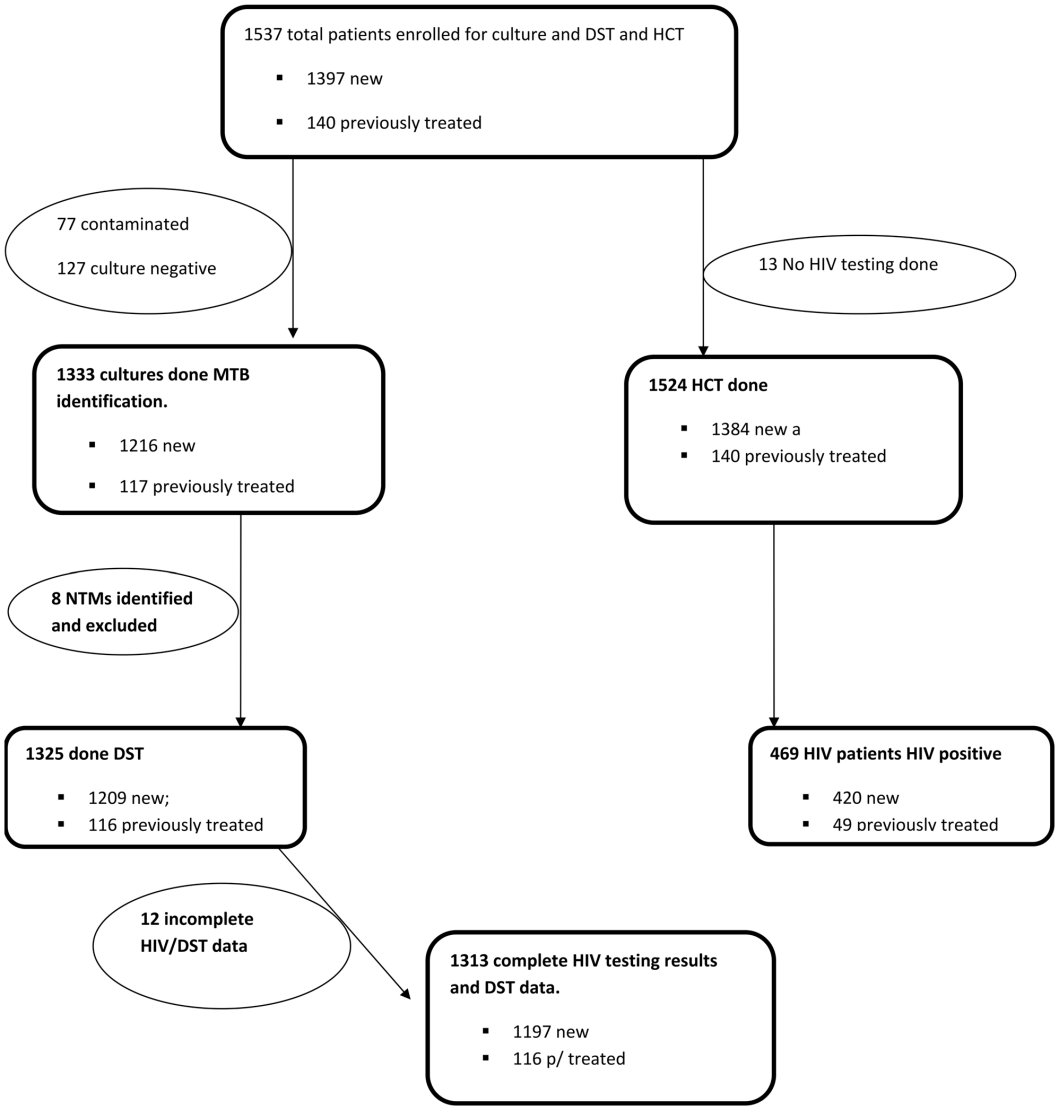
Flow Chart of patient enrollment in the National Anti-TB drug resistance survey in Uganda; December 2009–February 2011. Figure legend: **NTM = Non Tuberculous Mycobacteria.

Of the 1537 patients enrolled at 44 health facilities, 1397 (90.7%) were new and 140 (9.3%) previously treated (fig. 2). Enrollment rate for the new sputum smear positive cases was 93.1% (1397/1500). Nine of the 30 clusters failed to enroll the required 50 new sputum smear positive patients due to insufficient number of patients registered during the enrollment period. A total of 1018 (66.2%) patients were male and the median age of the enrolled patients was 34.6 years. The national coordination team re-interviewed 130 (8.3%) patients to confirm their treatment history and the categorization of the patients as new or retreatment by the facilities was found to be completely accurate.

### Culture Results, Table 1

Of 1537 enrolled patients, both LJ slants from 77 (5.0%) were contaminated leaving. 1460 patients who had culture results that were included in analysis. Of these, 127 (8.7%) were negative while 8 isolates grew non-tuberculous mycobacteria (NTM). A total of 1325 (90.5%) patients with culture-positive isolates underwent DST including 1209 isolates from new and 116 from previously treated patients. There was no statistically significant difference with respect to age, sex, history of previous treatment, and patient HIV status between patients who had culture- positive results and patients who had negative/contaminated cultures.

**Table 1:** Demographic Characteristics of Patients enrolled in National anti-TB drug resistance survey in Uganda; December 2009–February 2011.

Characteristic		N (%) N = 1537	Included for DST n (%) N = 1325	Not included for DST n (%) N = 212	p-value
**Sex**	Male	1018 (66.2)	874 (66.5)	144 (67.9)	0.69
	FemaleMissing	507 (33.0)12 (0.78)	439 (33.5)12 (0.91)	68 (32.1)–	
**Age**	13–14	33 (2.2)	30 (2.30)	3 (1.42)	0.23
	15–24	299 (19.5)	263 (19.9)	36 (17.0)	
	25–34	521 (33.9)	456 (34.4)	65 (30.7)	
	35–44	366 (23.8)	309 (23.3)	57 (25.9)	
	45–54	167 (10.9)	142 (10.7)	25 (11.8)	
	>55	139 (9.04)	113 (8.5)	26 (12.3)	
	Missing	12 (0.78)	12 (0.91)	0	
**HIV results**	Positive	469 (30.5)	399 (30.1)	70 (33.0)	0.28
	Negative	1056 (68.7)	914 (69.0)	142 (66.9)	
	Missing	12 (0.78)	12 (0.91)	0	
**Previous history of TB treatment**					
	Yes	140 (9.1)	116 (8.7)	24 (11.3)	0.19
	No	1385 (90.1)	1197(91.3)	188 (88.7)	
	Missing	12 (0.78)	12 (0.91)		
		N = 140	N = 116		
**Outcome of previous treatment**	successful	53 (37.9)	44 (37.9)	9 (37.5)	0.97
	unsuccessful	8762.1)	72(62.1)	15 (62.5)	
	unknown		–		
**Homeless**	yes	2 (0.1)	2 (0.1)	–	–
**Prisoner**	Yes	7	4	3	–

p-values for the patients included and those not included in the analysis of drug resistance.

### Drug Resistance Prevalence, Table 2

Among 1209 enrolled new patients with DST data, the prevalence of resistance to any of the drugs was 10.3% (n = 124; 95% (CI); 8.6–12.1). Any resistance to isoniazid was found in 60 (5.0%; 95% CI; 3.8–6.3), and to rifampicin in 23 (1.9%; 95% CI; 1.2–2.8) of the isolates, while 17 (1.4%; 95% CI; 0.80–2.2) showed MDR-TB. Monoresistance to rifampicin was observed in 3 (0.24%) of the isolates.

**Table 2:** Anti- tuberculosis Drug Resistance Among New and Previously Treated Sputum Smear- Positive TB patients in Uganda; December 2009–February 2011.

	New cases		Previously Treated		All cases	
Pattern of Resistance	Number (%)	95% CI	Number (%)	95% CI	Number (%)	95% CI
Total patients	**N = 1209**		**N = 116**		**1325**	
Susceptible to all	1085 (89.7)	87.9–91.3	86 (74.1)	65.2–81.8	1171 (88.4)	86.5–90.0
£Any Resistance	124 (10.3)	8.40–12.3	30 (25.9)	18.1–34.8	154 (11.6)	9.9–13.4
**Any Resistance to;**						
RMP	23 (1.9)	1.2–2.8	14 (12.1)	6.80–19.4	37 (2.80)	1.9– 3.8
INH	60 (5.0)	3.8–6.3	27 (23.3)	15.9–32.1	87 (6.56)	5.2–8.0
EMB	25 (2.1)	1.3–3.0	13 (11.2)	6.10–18.4	38 (2.90)	2.0–4.0
SM	76 (6.3)	4.9–7.8	20 (17.2)	10.6–25.3	96 (7.24)	5.9–8.7
**INH+RMP Resistant** (MDR)						
INH+RMP (Only)	3 (0.25)	0.0–0.6	2 (1.72)	0.2–6.1	5 (0.38)	0.1–0.8
INH+RMP+ EMB	0 (0)	–	0 (0)	–	0 (0)	–
INH+ RMP+ SM	6 (0.5)	0.0–1.0	3 (2.6)	0.5–7.3	9 (0.68)	0.3– 1.2
INH+RMP+EMB+SM	8 (0.70)	0.1–1.3	9 (7.8)	4.0–14.2	17 (1.30)	0.7–2.0
**All INH+RMP Resistant (MDR)**	**17 (1.40)**	**0.6–2.2**	**14 (12.1)**	**6.80–19.40**	**31 (2.3)**	**1.5–3.3**
INH+ Other Resistance						
INH+EMB	5 (0.38)	0.0–0.8				
INH+ SM	12 (0.9)	0.4–1.4	6 (5.2)	1.9–10.0	18 (1.4)	0.8–2.1
INH+EMB+ SM	0 (0)	–	0 (0)	–	(0)	–
RMP+other Resistance						
RMP+ EMB	0 (0)	–	0 (0)	–	0 (0)	–
RMP+ SM	2 (0.21)	0.1–0.6	0 (0)	–	2 (0.2)	0–0.5
RMP+ EMB+ SM	0 (0)	–	0 (0)	–	0 (0)	–
¥ **Mono Resistance to**;						
RMP	3 (0.24)	0.0–0.7	0(0)	–	4 (0.30)	0.0–0.7
INH	26 (2.1)	1.1–3.1	7 (6.0)	2.40–12.0	33 (2.5)	1.7–3.5
EMB	11 (0.9)	0.4–1.4	1 (0.9)	0.0–4.0	12 (0.9)	0.5–1.5
SM	47 (3.8)	2.7–5.0	2 (1.7)	0.2–6.1	49 (3.7)	2.7–4.8
**Other Resistance**						
EMB+ SM	1(0.05)	0–0.2	0 (0)	–	1 (0.1)	0–0.4

± RMP = rifampicin INH = isoniazid EMB = ethambutol SM = streptomycin.

£ Any Resistance: Resistance to any of the anti TB drugs either in combination or as single drug.

¥ Mono Resistance; Resistance to only one anti-TB drug.

Of the 116 previously treated patients, 53 (38.6%) had been cured, 58 (41.4%) completed treatment, 17 (12.1%) defaulted, 2 (1.4%) were treatment failures, while treatment outcomes of 9 (6.4%) patients were unknown. Thirty one (25.9%, 95% CI; 18.1–34.8) showed resistance to at least one drug. Any resistance to isoniazid was observed in 27 (23.3%, 95% CI; 15.3–32.0) and to rifampicin in 14 (12.1%; 95% CI, 6.7–19.4) patients. All the 14 (12.1%, 95% CI; 6.8–19.4) isolates resistant to rifampicin were MDR.

Overall the prevalence of any resistance and MDR when new and previously treated patients are combined was 11.6% (n = 154 95%; CI, 9.90–13.4) and 2.3% (n = 31; 95% CI 1.5–3.3) respectively. Of the 31 MDR-TB cases 17 (54.8; 95% CI 36.0–72.6) were resistant to all the four first line drugs. We found monoresistance prevalence highest for streptomycin 3.7% (n = 49; 95% CI 2.7–4.8) and lowest for rifampicin 0.3% (n = 4; 95% CI, 0–0.7).

Out of the 73 samples sent to the SRL for external QA, accuracy was 97.3% (n = 71) for isoniazid, rifampicin, and streptomycin and 95.8% (n = 70) for ethambutol. All MDR-TB cases were correctly identified with exception of one isolate that turned out to be pan-susceptible on retesting.

### Factors Associated with Drug Resistance, Table 3

Isolates from patients previously exposed to anti-TB drugs were more likely to show anti-TB drug resistance (odds ratio (OR) 9.02; 95% CI; 3.4–23.3 p<0.001). In multivariate analysis we found that patients enrolled in urban clusters were more likely to have MDR-TB, compared to those from rural clusters (Adjusted OR 6.0; 95%CI 1.40–25.3; p = 0.02). We also found a significant association between age and drug resistance; those >35 years were more likely to have MDR-TB as compared to patients <35 years among new patients (OR 2.0; 95% CI; 1.0–4.30), while among the previously treated patients this association was not significant (OR = 1). No other associated factors were identified. Of the 1537 patients enrolled, 1524 (99.1%) had HIV testing of whom 469 (30.7%, 95% CI; 28.4–33.1) tested positive. Among the 1313 patients with complete HIV and DST results, no significant association was observed between HIV infection and any resistance (OR 1.2, 95% CI; 0.8–1.7 p = 0.38), isoniazid resistance (OR 1.2; 95%CI 0.76–2.1 p = 0.36) or MDR (OR 1.5; 95%CI, 0.52–2.5; p = 0.71)in a multivariate analysis.

**Table 3:** Analysis of factors associated with Multi drug resistance in Uganda; December 2009–February 2011.

Risk Factor			Univariate	Multivariate
		N (%)	OR (95%CI)	OR (95% CI)
Sex				
	Male	21/881 (2.4)	0.94 (0.4–2.0)	1.2 (0.50–3.30)
	Female	10/444 (2.3)		
Age group (years)				
	<35	9/757 (1.2)		
	≥35	22/568 (3.9)	3.3 (1.5–7.0)	2 (1.0–4.3)
Residence	Urban	28/798 (3.51)	6.3 (1.9–20.9)	6.0 (1.44–25.3)
	Rural	3/527 (0.57)		
Previous history of TB treatment	Yes	14/116 (12.1)	8.6 (4.3–16.9)	8.6 (4.0–18.2)
	No	17/1209 (1.4)		
**HIV Status	Positive	11/388 (2.8)	1.3 (0.6–2.6)	
	Negative	20/984 (2.2)		

Variable included in the multivariate model were, age, sex, residence and previous history of TB treatment.

** Analysis limited to univariate level as inclusion at multivariate level masked the apparent association between MDR and potential risk factors.

### XDR Prevalence

All the 31 MDR isolates were tested for susceptibility to kanamycin and ofloxacin to which all demonstrated complete susceptibility showing absence of XDR among the study patients.

## Discussion

This study is the first nationally representative anti-TB drug resistance survey in Uganda and one of the studies done in Sub Saharan Africa at a national scale. The survey showed an MDR-TB prevalence of 1.4% and 12.1% among new and previously treated sputum smear-positive TB patients respectively. Since settings with an MDR-TB prevalence of less than 3% among new patients are classified as having a low MDR-TB burden, [22] we conclude that the prevalence of MDR-TB among new smear positive patients in Uganda is low. MDR-TB among previously treated TB cases however was moderately high (12.1%). The prevalence of resistance to any of the first line anti-TB drugs, among new (8.3%) and previously treated (25.9%) patients was consistent with findings of a recent community based survey in Kampala city as shown in our previous report [16]. Other nationwide surveys in the region have observed the prevalence in the same range. The prevalence of any resistance among new and previously treated patients was 8.3% and 20% respectively in the United Republic of Tanzania [23]. A related survey done in Rwanda showed an MDR-TB rate of 3.9% when new and previously treated patients were combined as compared to the 2.3% that we report for the new and previously treated patients together in this survey [24].

While it’s difficult to directly compare outcomes from different countries, especially when surveys are conducted at different time periods, the data from this survey show that levels of MDR-TB among newly diagnosed smear positive TB patients in Uganda are relatively low. This could potentially be attributed among other things to the limited use of rifampicin only during the first 2 months (2EHRZ/6EH) for new TB cases who contribute over 90% of the disease burden, assuming a good adherence to TB therapy. This is contrary to the earlier reports that shorter duration of rifampicin may lead to increase in acquired resistance [25]. However, we also acknowledge the lower treatment success rate (70%) among new smear positive patients and the potential role of a rifampicin lacking TB regimen for this lower success rate [26]. In addition to the 17 cases identified as MDR-TB, an additional 43 and 6 new smear positive cases were found to have resistance to isoniazid and rifampicin respectively thus placing these patients just one step away from developing MDR-TB. If the national TB program plans to adopt 6 month TB regimen with 4 months of rifampicin during the continuation phase in the near future, directly observed therapy and adherence to therapy for all TB cases especially new TB cases has to be carefully monitored and completion ensured. The higher rates among previously treated TB patients as we see in this study have been attributed to stepwise selection of mutants due to drug resistance conferring genes [27]. Higher levels of MDR-TB (12.1%) and resistance to any drug (25.3%) among previously treated patients raises concerns about the quality of directly observed therapy and adherence to treatment. XDR-TB was not detected among the survey participants. Our study was not powered to assess the prevalence of XDR-TB among the study participants so no definitive conclusions could be made about the prevalence of XDR-TB in the country. However, XDR-TB might be a very limited problem if at all in Uganda especially given the limited use and availability of the second line drugs. Disaggregated by age, the older age group (>35 years) had higher levels of MDR-TB than the young age group (OR 2.0; 95% CI; 1.0–4.30) implying higher chances of exposure over time to drug-resistant TB in the community by the older than the young population. Patients diagnosed in urban clusters were more likely to have MDR-TB (OR = 6; 95% CI, 1.44–25.3 ) than those from rural facilities, probably as a result of referral of complicated TB cases including MDR-TB suspects from rural health units to referral centers (regional/district hospitals) commonly located in urban areas. Overcrowding in the towns and cities might also have facilitated primary MDR-TB transmission resulting in majority of the cases being in the urban clusters.

HIV prevalence was 30.7% (95% CI; 28.4–33.1) among study participants was lower than the 54% found among all TB cases through the TB surveillance system [1]. HIV was more prevalent among female participants than among males (OR 1.89; 95%; CI 1.45–2.50; p = <0.01) and this finding was consistent with the gender wise HIV prevalence in the general population. According to the recent AIDS Indicator Survey (AIS) the HIV prevalence among females aged 15–59 was 7.6% and 5.6% among men of the same age group [28]. Like in other published studies, there was no statistically significant association between HIV infection and MDR-TB [29]–[32], although some studies have reported contrasting findings in which such an association has been documented [33].

Of major concern among the findings is the existence of primary resistance (rifampicin 1.9%, isoniazid 5%, streptomycin 6.3%) implying ongoing transmission of drug resistant strains in the community. This could imply weakness in infection control measures which should therefore be strengthened through dissemination of TB infection control guidelines by the NTLP. Priority should also be accorded to TB infection control training for health care workers in the TB diagnostic and treatment centers especially those which offer comprehensive TB/HIV care. Health care work was identified as a risk factor for resistance to any of the anti-TB drugs according to our earlier report [16], suggesting that nosocomial transmission of drug-resistant TB strains occurs.

### Limitations

The survey only represented patients diagnosed through the NTLP-supervised health facilities and does not account for drug resistance patterns among population not having access to the health system and, we did not have data about the size and characteristics of this patient population. Although the survey was conducted using the most recent WHO guidance, smear negative patients were not included in the survey. Our findings thus might not account for potentially different drug resistance pattern among smear negative TB patients. Moreover, inclusion of smear negative patient who are more likely to be HIV positive might impact the association between MDR-TB and HIV status. Nosocomial transmission in congregate settings has been proven to be one of the major risk factors for transmission of MDR-TB. We were not able to assess it during this survey.

Also, the sampling frame was based on TB case notification in 2005 in Uganda, and a number of changes in health care delivery system had taken place since, especially the establishment of new districts and new health facilities, which did not make part of the sampling frame but shared the patients with the included facilities. Incidents of untimely closure of the local courier system in some parts of the country might have led to delayed or non-delivery of the sputum samples from these clusters which could have contributed to the observed contamination and enrollment rates that varied from expected. However to avoid selection bias due to unequal participation rates, we controlled for this occurrence at the analysis level by weighting for the exact sampling probabilities for each individual patient for whom DST results were available across the clusters. We could have done multiple imputation for the missing drug resistance results, but with the amount of data that were available to predict drug resistance status for missing results, this method would most likely lead to biased results as well. The distances covered to reach the nearest diagnostic/treatment units (DTUs) in some clusters were too long to bring the early morning sample after submission of the spot sample as the study required. Some patients therefore failed to deliver the early morning sample within the required period. These numbers were however too small to affect the enrollment rates and the occurrence was too random to result into any bias that could significantly affect our results. Our conclusions about XDR-TB prevalence were based on resistance studies against kanamycin alone although cross resistance with other injectable second line anti-TB drugs has been documented.

### Conclusion and Recommendations

Anti-TB drug resistance among new smear positive TB cases was low and not associated with HIV infection in Uganda, despite the high TB-HIV co infection rates. We therefore recommend that strengthening and implementation of appropriate interventions is critical to keep MDR-TB levels low in the country or to reverse the trends. The NTLP needs to focus on improving the quality of directly observed therapy and develop interventions to support patient adherence in order to prevent development of acquired resistance. The NTLP should strengthen the existing specimen referral system and implementation of a routine surveillance system for anti-TB drug resistance to follow drug resistance trends over time and to identify outbreaks of drug resistant TB. Establishment of an effective MDR-TB control program and treatment strategy would be critical for effective clinical management of all cases of drug resistant TB. The introduction of rapid molecular diagnostic tests like Xpert MTB/RIF present a unique opportunity to diagnose MTB and identify rifampicin resistance within 2 hours [34], [35]. WHO recommends the use of Xpert MTB/RIF as the first diagnostic test for persons at risk of developing MDR-TB and among people living with HIV [36] and NTLP should consider targeted roll out this technology. Efforts towards TB infection control including ensuring adequate ventilation for inpatient wards and outpatient waiting areas, provision of protective wear for patients and most importantly effective treatment of drug susceptible cases should be ensured to minimize emergence of new MDR-TB cases. We recommend further studies to establish whether MDR-TB cases are due to reactivation of latent disease or transmission of new infections and whether there exists predominance of a particular MTB strain among drug resistant patients as described elsewhere [37].

## References

[1] WHO (2011) Global Tuberculosis Control: Surveillance, planning, financing: WHO report. in WHO/HTM/TB/2011.

[2] Corbet EL, Watt CJ, Walker N, Maher D, Williams BG, et al. (2003) The growing burden of tuberculosis: global trends and interactions with the HIV epidemic. Archives of Internal Medicine 2003. 163(9): p 1009–21. 10.1001/archinte.163.9.1009 12742798

[3] Pooran A, Pieterson E, Davids M, Theron G, Dheda K (2013) What is the cost of diagnosis and management of drug resistant tuberculosis in South Africa. PLOS ONE, 8(1). 10.1371/journal.pone.0054587 PMC3548831 23349933

[4] Cocker RJ (2004) Review: Multidrug-resistant tuberculosis: public health challenges. Tropical Medicine and International Health 9(1): p 25–40. 10.1046/j.1365-3156.2003.01156.x 14728604

[5] WHO (2010) Multidrug and extensively drug-resistant TB (M/XDR-TB): 2010 global report on surveillance and response. Geneva Switzerland.

[6] Wright A, Zignol Matteo, Van Deun A, Falzon D, Gerdes SR (2009) Epidemiology of antituberculosis drug resistance 2002–07: an updated analysis of the Global Project on Anti-Tuberculosis Drug Resistance Surveillance. Lancet 373: p. 1861–1873. 10.1016/S0140-6736(09)60331-7 19375159

[7] Lambregts-van Weezenbeek CSB, Veen J (1995) >Control of drug resistant tuberculosis. Tubercle Lung Dis, 76: p 455–459. 10.1016/0962-8479(95)90014-4 7496009

[8] Surveillance (2001) W.H.O.-I.U.A.T.a.L.D.W.G.o.A.-t.D.R., New England Journal of Medicine, 344(17): p. 1294–303. 10.1056/NEJM200104263441706 11320389

[9] Dupon M, Leroy V, Sentilhes A, Pellegrin JL (1995) Tuberculosis and HIV infection: a cohort study of incidence and susceptibility to antituberculous drugs, Bordeaux, 1985–1993. Grouped’Epidemiologie Clinique du SIDA en Aquitane. AIDS, 9(6): p. 577–83. 7662196

[10] WHO (2011) Towards universal access to diagnosisand treatment of multidrug-resistant and extensively drug-resistanttuberculosis by 2015, in WHO/HTM/TB/2011 Switzerland.

[11] Wells CD, Cegielski JP, Nelson LJ, Laserson KF, Holtz TH, et al. (2007) HIV Infection and Multidrug-Resistant Tuberculosis-The Perfect Storm. Journal of Infectioius Diseases 196: p. S86–S107. 10.1086/518665 17624830

[12] Sandman L, Schluger NW, Davidow AL, Bonk S (1999) Risk factors for rifampin-monoresistant tuberculosis: a case-control study. American Journal of Respiratory and Critical Care Medicine, 159(2): p. 468–472. 10.1164/ajrccm.159.2.9805097 9927359

[13] WHO ed (2013) Guidelines for surveillance of drug resistance in tuberculosis 2nd ed. 18–19.

[14] Bretzel G, Zignol Matteo A, Wendl-Richter U, Aisu T (1999) Antituberculosis drug resistance surveillance in Uganda 1996–1997. Int J Tuberc Lung Dis, 3: p. 810–815. 10488890

[15] Joloba ML, Whalen CC, Cave DM, Eisenach KD, Johnson JL (2000) Determination of drug susceptibility and DNA fingerprint patterns of clinical isolates of Mycobacterium tuberculosis from Kampala, Uganda. East African medical journal, 77(2): p. 111–5. 10774085

[16] Lukoye D, Cobelens FG, Ezati N, Kirimunda S, Adatu FE, et al (2011) Rates of Anti-Tuberculosis Drug Resistance in Kampala-Uganda Are Low and Not Associated with HIV Infection. PLoS ONE 6(1): e16130. 21249225 10.1371/journal.pone.0016130 PMC3018425

[17] WHO (2003) Guidelines for surveillance of drug resistance in Tuberculosis.GenevaSwitzerland, in Publication. WHO/TB/320.

[18] WHO ed (2009) Guidelines for surveillance of drug resistant tuberculosis 2009.

[19] MOH (2010) Manual of National Tuberculosis and Refernce Program, NDC, Editor M.O.H: Kampala. 60.

[20] Van Embden JD, Cave MD, Crawford JT, Dale JW, Eisenach KD (1993) Strain identification of Mycobacterium tuberculosis by DNAfingerprinting: recommendations for a standardized methodology. J ClinMicrobiol 31: 406–409. 10.1128/jcm.31.2.406-409.1993 PMC262774 8381814

[21] Rao JN, Scott AJ (1992) A simple method for the analysis of clustered binary data. p. 577–85. 1637980

[22] WHO/IUATLD (2008) Global Project on Anti-tuberculosis Drug Resistance Surveillance;WHO/IUATLD, Editor.

[23] Chonde TM, Basra D, Mfinanga SG, Range N, Lwilla F, et al. (2010) National anti-Tuberculosis drug resistance study in Tanzania. Int. J. Turberc Lung Dis, 14(8) p. 967–972. 20626940

[24] Umubyeyi AN, Vandabriel G, Gasana M, Basinga P, Zawadi JP, et al (2007) *Results of a national survey on drug resistance among pulmonary tuberculosis patients in Rwanda.* int j TUBERC LUNG DIS, 1: p. 189–194. 17263290

[25] Menzies D, Benedetti A, Paydar A, Martin I, Royce S, et al. (2009): Effect of duration and intermittency of rifampicin on tuberculosis treatment outcomes. A systemic review and meta analysis. PLoS Med. 6 e1000(146 ). 10.1371/journal.pmed.1000146 PMC2736385 19753109

[26] Jindani A, Nunn A, Enarson DA (2004) Two 8-month regimens of chemotherapy for treatment of newly diagnosed pulmonary tuberculosis: international multicentrerandomised trial. Lancet. 8(364): p. 1244–51. 10.1016/S0140-6736(04)17141-9 15464185

[27] Lew W, Pai M, Oxlade O, Martin D, Menzies D (2008) Initial drug resistance and tuberculosis treatment out comes; systemic review and meta analysis. Ann Intern. MED. 149: 123–134. 10.7326/0003-4819-149-2-200807150-00008 18626051

[28] MOH (2011) Uganda AIDS Indicator Survey (AIS), N.D.C, Editor. 2010, Ministry Of Health: Kampala. 25.

[29] Chum HJ, O’Brien RJ, Chonde TM, Graf P, Rieder HL, et al (1996) 1991–1993 Aids., An epidemiological study of tuberculosis and HIV infection in Tanzania *1991–1993.* Aids, 10. 10.1097/00002030-199603000-00009 8882670

[30] Anastasis D, Pillai G, Rambirititch V, AbdoolKarim SS (1997) A retrospective study of human immune deficiency virus infection and drug resistant tuberculosis in Durban South Africa. 1: 220–224. 9432367

[31] Murry J, Sonnenberg P, Shearer S, Godfrey Faussett P (2000) Drug resistant tuberculosis in a cohort of South African goldminers with high prevalence of HIV infection. S. Afr Med J 90: 381–386. 10957924

[32] Kenyon TA, Mwasekaga MJ, Huebner R, Rumisha D, Binkin N, et al (1999) Low levels of anti-tuberculosis drug resistance amidst rapidly increasing tuberculosis and human immunodefiency virus epidemics in Botswana. Int J Tuberc Lung Dis 3: 4–11. 10094163

[33] Patel KB, Belmonte R, Crowe HM (1995) Drug malabsorption and resistant tuberculosis in HIV infected patients N Eng. J Med 332: 336–337. 10.1056/NEJM199502023320518 7816080

[34] Yoon C, Cattamanchi A, Davis JL, Worodria W, den Boon S, et al. (2012) Impact of Xpert MTB/RIF Testing on Tuberculosis Management and Outcomes in Hospitalized Patients in Uganda. PLoS ONE, 7(11). 10.1371/journal.pone.0048599 PMC3490868 23139799

[35] Lawn SD, Nicol MP (2011) Xpert(R) MTB/RIF assay; development, evaluation and implementation of a new rapid molecular diagnostic for tuberculosis and rifampicin resistance. Future Microbiol 6(9): 1067–1082. 21958145 10.2217/fmb.11.84 PMC3252681

[36] WHO (2011) Rapid implementation of the Xpert MTB/RIF diagnostic test: technical and operational “How-to” practical considerations 8.

[37] Cox HS, Kubica T, Doshetov D, YKebede Y, Rüsch-Gerdess S, et al (2005) The Beijing genotype and drug resistant tuberculosis in the Aral Searegion of Central Asia. Respiratory Research (6): 134. 16277659 10.1186/1465-9921-6-134 PMC1299328

